# The Impact of Transportation Restructuring on the Intensity of Greenhouse Gas Emissions: Empirical Data from China

**DOI:** 10.3390/ijerph191912960

**Published:** 2022-10-10

**Authors:** Huiling Wang, Jiaxin Luo, Mengtian Zhang, Yue Ling

**Affiliations:** School of Economics, Lanzhou University, Lanzhou 730000, China

**Keywords:** transportation restructuring, input-output analysis, emission intensity, greenhouse gases, climate change

## Abstract

Adjusting transportation structure to reduce the intensity of greenhouse gas emissions is an effective way to address climate change issues. This paper selects six transport sectors and constructs a hybrid input-output model to study the impact of transportation restructuring on the intensity of CO_2_ and non-CO_2_ greenhouse gas emissions in each sector during different periods. The results show that the effect of transportation restructuring on greenhouse gas emissions is manifested differently in different time periods. After 2008, transportation restructuring had a significant effect on reducing the intensity of greenhouse gas emissions in all sectors. However, the impact of transportation restructuring on the intensity of non-CO_2_ greenhouse gas emissions is limited. It is also found that the railway transport sector has been a low-impact transport sector in terms of greenhouse gas emissions since 2004, which provides insights for the optimization of China’s transportation structure.

## 1. Introduction

Climate change has become a key nontraditional international security issue. Excessive greenhouse gas (GHG) emissions are one of the main causes of increased climate problems [1]. Climate change will not only affect population health directly by increasing the frequency and intensity of heat, drought, and heavy rainfall, but also indirectly by increasing air pollution, accelerating the spread of disease vectors, and affecting food security and mental health [2]. Reducing GHG emissions can provide a fundamental solution to climate change while delivering significant population health synergies [2,3]. Therefore, the need to reduce GHG emissions and develop a low-carbon economy has become a global consensus [4]. Currently, 38 economies have formally proposed timelines for achieving their carbon neutrality goals. On 22 September 2020, Chinese President Xi Jinping solemnly declared at the general debate of the 75th UN General Assembly that “we will work toward achieving carbon neutrality by 2060”. Carbon neutrality refers to offsetting the total amount of GHG emissions produced directly or indirectly by an enterprise or individual during a certain period through energy savings and reforestation to achieve net zero carbon emissions. Based on this, we can see that the goal of “carbon neutrality” can only be achieved through both emission reductions and biological carbon sequestration. On the one hand, international experience has shown that transport, as the second largest GHG emitting sector in the world [5], is probably the sector in which it is most difficult to reduce GHG emissions [6]. In China, the transportation sector already contributes more than 10% of the country’s total carbon emissions [7]; on the other hand, there is great potential for reducing emissions within the transport sector, with Wang et al. [8] predicting a potential GHG reduction of up to 40 million to 250 million tons in China’s transport sector over the next decade. Therefore, studying the impact of the development of the transportation industry on GHG emissions is of urgent practical importance for achieving carbon neutrality, improving national health, and ensuring the high-quality development of China’s economy. In addition, studying this issue can provide a reference for countries around the world as they combat the problem of excessive GHG emissions.

The transportation structure is particularly critical when studying the impact of transport development on GHG emissions [9,10,11]. On the one hand, the intensity of energy consumption varies across transport sectors, and the composition of the transport sector (i.e., the transportation structure) determines the intensity of energy consumption in the transportation industry, which in turn directly affects the GHG emission intensity of the transportation industry. On the other hand, the excessive emission of GHGs affects the human living environment and based on the pursuit of a healthy living environment, the transportation structure should be optimized with the goal of reducing GHG emissions [12]. Therefore, studies of the impact of the development of the transportation industry on GHG emissions must be placed in the broader context of transportation restructuring.

The transportation structure refers to the ratio and composition of interconnections and links within and outside the transport sector, i.e., the share of different transportation modes within the transportation industry [13]. It reflects the specific share of total transport turnover in a region belonging to different modes of transport. Transportation restructuring stems from changes in the industrial structure [14]. Economic development leads to the optimization of the industrial structure, which in turn leads to the adjustment of the transportation structure [13]. Because the industrial structure determines the intensity of resource consumption by an economic unit [15], the restructuring of the transportation industry driven by the optimization of the industrial structure greatly affects the deep decarbonization process within the transportation industry [16], as well as the GHG emission intensity of each transport sector. Transportation restructuring is essentially the process of fully utilizing the comparative advantages of the various modes of transportation. In this process, passengers begin to prioritize the development of public transport and discourage the excessive use of private cars [17]. On the freight side, the capacity structure is optimized to promote larger transport vehicles with trailers, a process that is accompanied by measures to improve the road network and raise the relevant technical levels of the workers in the industry. These improvements reduce the intensity of GHG emissions in each transportation sector. Moreover, as different modes of transportation have different costs and transport volumes, transportation restructuring also affects the output of other related sectors, which in turn affects the intensity of GHG emissions in those other sectors. In terms of empirical evidence, the academic community has not yet reached a consensus. Some empirical results confirm that transportation restructuring can reduce GHG emissions intensity and emissions from the transportation industry [9,18,19,20,21,22,23]. However, some scholars have found that transportation restructuring has increased GHG emissions intensity and emissions from the transportation industry [24,25,26]. It takes a relatively long time for transportation restructuring to have an impact on the various industrial sectors, so research results are dependent on the period of study [27]. In addition, a review of the literature reveals that scholars have devoted more effort to studying the impact of transportation restructuring on the level of GHG emissions, and fewer have studied its impact on emission intensities [28]. GHG emission intensity is a quality indicator for reducing GHG emissions while ensuring economic growth [29]. Therefore, given the current development of China’s transportation industry, this paper selects six transportation sectors—namely, the railway, highway, domestic public transport (The domestic public transport sector includes passenger bus transport, urban rail transport, cab passenger transport, city ferries, and other urban public transport.), water, air, and pipeline sectors (hereafter “the six transportation sectors”)—and chooses the period of 1990–2016, 27 years of data, to study the impact of transport restructuring on the intensity of GHG emissions in the transport and non-transport sectors in stages.

A GHG is any gas that absorbs and releases infrared radiation and exists in the atmosphere [30]. However, some scholars tend to equate GHGs with CO_2_ and have ignored non-CO_2_ GHGs. China has emphasized, “controlling non-CO_2_ GHG emissions” in its 13th Five-Year Plan. Although emitted into the atmosphere far less than CO_2_, non-CO_2_ GHGs have high global warming potential, short life cycles, and low abatement costs relative to CO_2_ [31]. Because of these characteristics, non-CO_2_ GHGs have greater mitigation potential and should not be ignored in climate change research. As a result, this paper divides GHGs into CO_2_ and non-CO_2_ GHGs and examines each separately.

The Intergovernmental Panel on Climate Change (IPCC) has proposed two approaches to measuring carbon emissions in the transportation industry. One approach is to calculate carbon emissions based on energy consumption during transportation and the corresponding carbon emission coefficients [20,32], which is a “top-down” approach. The other is to estimate energy consumption by the distance traveled and the transportation mode chosen and to then calculate the carbon emissions with the carbon emission coefficient for the energy type corresponding to the transportation mode [6,29,33,34,35,36], which is a “bottom-up” approach. The former cannot distinguish the impacts of the different transport sectors on GHG emissions; the latter is prone to errors in the final results because some data need to be estimated. In either case, both methods can only measure the direct GHG emissions from the transport sector and neglect the indirect GHG emissions caused by the transport sector, which leads to errors in the final results. However, input-output modeling is a powerful tool for examining the interaction of various sectors of the national economy. Leontief [37] began by adding an environmental pollution module to the input-output table and then created a hybrid input-output table to investigate the environmental impact of economic structure. This serves as a guide for calculating the unit emissions of GHG from each transportation sector, including direct and indirect emissions. The noncompetitive input-output tables and environmental extension tables provided by the EORA database can provide accurate CO_2_ and non-CO_2_ GHG emissions for each industrial sector. Therefore, a hybrid input-output table can enable researchers to accurately obtain the intensity of GHG emissions for each sector containing direct and indirect. On the other hand, since the input-output table published by the Chinese Bureau of Statistics every five years is competitive and the production of imported products occurs abroad, the corresponding energy consumption and GHG emissions also occur abroad. An empirical study that directly uses this competitive input-output table is likely to overestimate the GHG emissions caused by the final demand for each product. Therefore, in this paper, the noncompetitive Chinese input-output tables provided by the EORA database are used to study the impact of transportation restructuring on the intensity of CO_2_ and non-CO_2_ GHG emissions.

The contributions of this paper are as follows: 1. This study constructs a hybrid input-output table to calculate the total direct and indirect GHG emission intensity of the six transportation sectors in China from 1990–2016. 2. This study could fill the gap in the literature on the impact of transport restructuring on GHG emission intensity, especially non-CO_2_ GHG emission intensity. 3. By defining the GHG influence coefficient, transportation sectors with low impact on GHG emissions were screened. 4. This study examines the impact of transportation restructuring on the intensity of CO_2_ and non-CO_2_ greenhouse gas emissions from non-transport sectors.

## 2. Methods

### 2.1. Structure of a Hybrid Input-Output Table

As shown in Table 1, a hybrid input-output table is an expansion of the GHG module found below the industrial sector in a traditional input-output table, with CO_2_ and non-CO_2_ GHGs selected as the subsectors within the GHG module. In this table, x_ij_ represents the value of the output from sector i that is consumed by production activities in sector j. y_i_ and X_i_ are the final use and the gross output of sector i, respectively, g_1j_ and g_2j_ represent the physical quantity of CO_2_ and non-CO_2_ GHG emitted by sector, and e_i_ and t_i_ represent the final emissions and gross emissions of the two GHG sectors. The monetary unit for this table is the US dollar, and the physical units are kilograms.

### 2.2. Noncompetitive Hybrid Input-Output Model

According to the equilibrium relationship that “total input = total output” assumed in the input-output table, the following can be obtained:

(1) $$\overset{n}{\underset{i=1}{\sum }}X_{i}=\overset{n}{\underset{j=1}{\sum }}X_{j}$$

(2) $$\sum_{i=1}^{n}x_{ij}+v_{j}=\sum_{j=1}^{n}x_{ij}+y_{i}\;(\mathrm{When}\;i=j)$$

The matrix form is as follows:

AX + Y = X(3)

(4) $$X={\left(I-A\right)}^{-1}Y$$

where X is the total output matrix for each industrial sector and Y is the final demand matrix for each industrial sector. *A* is the direct consumption coefficient matrix, and *I* is the unit matrix.

Similarly, the equilibrium relationship for the GHG module can be obtained as follows:

(5) $$\overset{n}{\underset{j=1}{\sum }}g_{ij}+e_{i}=t_{i}$$

The direct emission coefficients for GHGs were defined and modeled based on the direct consumption coefficients in the input-output tables.

The direct emission coefficient for GHGs is given by:

(6) $$c_{ij}=\frac{g_{ij}}{X_{j}}$$

This leads to a matrix of direct GHG emission coefficients *C*:

$$C=\left[\begin{array}{ccc} c_{11} & \cdots & c_{1n}\\ \vdots & \ddots & \vdots \\ c_{n1} & \cdots & c_{nn} \end{array}\right]$$

This leads to matrix expressions for the GHG module:

CX + E = T(7)

By substituting Equation (4) into Equation (7), the following is obtained:

G = C(I − A)^−1^Y (8)

If B^g^ is the complete emission coefficient for the GHG, it follows that:

B^g^ = C(I − A)^−1^ (9)

Since the products of each sector are directly linked to the relevant sectors in the production process, but also indirectly linked to some sectors, there are indirect emissions of greenhouse gases in the production process of the products of each sector in addition to the direct emissions of greenhouse gases. Equation (9) represents the quantitative relationship between GHG emissions and final demand, quantifying the total direct and indirect emissions of GHGs per unit of final product produced in sector j, i.e., the GHG emission intensity of sector j.

To further quantify the impact of transportation restructuring on the intensity of GHG emissions, the 123 sectors in China’s noncompetitive input-output table are divided into the transportation and the nontransportation sector; i.e., the six transportation sectors are excluded, and the remaining sectors are merged into the nontransportation sector. This study follows the model design of Yijun and Yuanyuan (2014) [38] to further decompose Equation (9). By allowing $B_{0}^{g}$ and $B_{1}^{g}$ to represent the complete emission coefficient matrices for the GHGs over two consecutive years and letting $A_{0},A_{1}$ and $C_{0},C_{1}$ be the direct consumption coefficient matrices and the direct emission coefficient matrices for the two consecutive years, respectively, we can define △$B^{g}$, △C, and △A as the differences in the matrix of complete emission coefficients, the matrix of direct emission coefficients and the matrix of direct consumption coefficients between two consecutive years. It follows that:

(10) $$△B^{g}=B_{1}^{g}-B_{0}^{g}$$

(11) $$△C=C_{1}-C_{0}$$

(12) $$△A=A_{1}-A_{0}$$

Multiplying both sides of the equation $B_{0}^{g}$= $C_{0}{\left(I-A_{0}\right)}^{-1}$ by $\left(I-A_{0}\right)$ it follows that:

$$B_{0}^{g}\left(I-A_{0}\right)=C_{0}{\left(I-A_{0}\right)}^{-1}\left(I-A_{0}\right)$$

(13) $$B_{0}^{g}\left(I-A_{0}\right)=C_{0}$$

Taking the first-order difference on Equation (10), it follows that:

(14) $$△B^{g}-B_{0}^{g}△A-△B^{g}A_{0}-△B^{g}△A=△C$$

Sorting for △$B^{g}$, it follows that:

$$△B^{g}-B_{0}^{g}△A-△B^{g}A_{0}-△B^{g}△A=△C$$

$$△B^{g}\left(I-A_{0}\right)-\left(B_{0}^{g}+△B^{g}\right)△A=△C$$

$$△B^{g}\left(I-A_{0}\right)-\left(B_{0}^{g}+B_{1}^{g}-B_{0}^{g}\right)△A=△C$$

$$△B^{g}\left(I-A_{0}\right)-B_{1}^{g}△A=△C$$

$$△B^{g}\left(I-A_{0}\right)=B_{1}^{g}△A+△C$$

$$△B^{g}\left(I-A_{0}\right){\left(I-A_{0}\right)}^{-1}=B_{1}^{g}△A{\left(I-A_{0}\right)}^{-1}+△C{\left(I-A_{0}\right)}^{-1}$$

(15) $$△B^{g}=B_{1}^{g}△A{\left(I-A_{0}\right)}^{-1}+△C{\left(I-A_{0}\right)}^{-1}$$

In Equation (15), $B_{1}^{g}$△A${\left(I-A_{0}\right)}^{-1}$ reflects the impact of transportation restructuring on the intensity of GHG emissions, and △C${\left(I-A_{0}\right)}^{-1}$ represents the impact of technological progress on the intensity of GHG emissions. The latter is beyond the scope of this paper and is not discussed further.

To quantify the degree of the impact of different transport sectors on GHG emissions, this study refers to the concept of the influence coefficient within the input-output model and proposes a GHG influence coefficient:

(16) rj=bjg1n∑j=1nbjg,j=1,2,3…,n

Equation (16) reflects the extent to which a one-unit increase in final demand in sector j changes the society-wide emission for a particular type of GHG. $r_{j}>1$ indicates that sector j has a greater impact on GHG emissions than the industry-wide average; conversely, $r_{j}<1$ indicates that sector j has a smaller impact on GHG emissions than the industry-wide average. A higher GHG influence coefficient indicates that this sector has a greater impact on GHG emissions, and vice versa.

### 2.3. Determination of the Study Period

It is clear from the above that the GHG emissions from the various transport sectors in China are closely related to China’s economic development and influenced by industrial restructuring. Therefore, the selection of the observation period for transportation restructuring needs to account for economic development and industrial structure changes. Changes in the transportation structure reflect changes in transportation demand [14], and the dynamics of the changes in transportation demand can be considered in terms of both passengers and freight. On the freight side, due to the different costs, energy consumption and freight volume in each transport sector and given the continued development of the economy, the very large demand for freight transport is expected to prompt a flow of factors of production toward transport sectors with low costs, low energy consumption, and high freight volume, such as the pipeline, water, and railway sectors, and thus promote the adjustment of the transportation structure. From the passenger side, with the increase in disposable income, people are expected to prefer to travel by modes of transport with high elasticities of demand, which in turn is expected to promote the development of the high-speed rail and air transport sectors, which have short passenger travel times and high levels of comfort but also high costs, ultimately promoting the restructuring of the transportation industry, i.e., the “Engel effect” within the transportation industry [39]. In addition, the development of international trade can also affect the transportation structure and increase GHG emissions from the transportation industry [40]. In 1990, the Seventh Plenary Session of the 13th CPC Central Committee proposed that “to give priority to the development of transportation and postal and telecommunications to meet the needs of national economic development and opening up to the outside world... In terms of transportation, we should focus on the construction of a comprehensive transportation system.” [41]. This proposal was approved by the Fourth Session of the Seventh National People’s Congress on April 9 of the following year [42]. Since then, the Chinese government has been guiding the restructuring of the transportation industry. Therefore, based on the intrinsic dynamics of China’s transportation restructuring, this paper sets 1990 as the first year of that transportation restructuring. Combining Jinglian ‘s [43] study of China’s economic reform and Lu and Xuehua’s [44] study of the evolution of China’s industrial structure, we choose three events to divide the period 1990-2016 into four transportation restructuring observation periods: the adoption of The Decision of the CPC Central Committee on Several Issues Concerning the Establishment of a Socialist Market Economy System, China’s formal accession to the World Trade Organization (WTO), and the 2008 global financial crisis. The period 1990–1994 is defined as Observation Period 1. Here, we study the impact of China’s transportation restructuring on the intensity of GHG emissions in the context of the linking of various modes of transportation and the optimization of the economic structure. The period 1994–2002 is defined as Observation Period 2. Here, we study the impact of China’s transportation restructuring on the intensity of GHG emissions in the context of comprehensive market-oriented reforms and active industrial restructuring. The period 2002–2009 is defined as Observation Period 3, where we study the impact of China’s transportation restructuring on the intensity of GHG emissions in the context of China’s accession to the WTO, the acceleration of economic development and the significant increase in transportation demand. Finally, the period 2009–2016 is defined as Observation Period 4, where we study the impact of China’s transportation restructuring on the intensity of GHG emissions in the context of the Chinese economy entering a “new normal” in the aftermath of the U.S. subprime mortgage crisis.

### 2.4. Data Processing

To study the impact of transportation restructuring on the intensity of CO_2_ and non-CO_2_ GHG emissions more precisely, the 1990–2016 noncompetitive input-output tables and environmental accounting data for sector 123 provided by the EORA database are used. In these data, the railway passenger transport and railway freight transport sectors are merged into the railway transport sector, and the air passenger transport and air freight transport sectors are merged into the air transport sector. In the GHG module, the emissions of methane (CH_4_), nitrous oxide (N_2_O), hydrofluorocarbons (HFCs), perfluorocarbons (PFCs), sulfur hexafluoride (SF_6_) and nitrogen trifluoride (NF3) are summed and combined into the non-CO_2_ GHG sector, referencing the six main non-CO_2_ GHGs set out in the Kyoto Protocol’s second commitment (2013).

## 3. Results

### 3.1. GHG influence Coefficient Analysis

#### 3.1.1. CO_2_ Influence Coefficient

**Figure 1:**
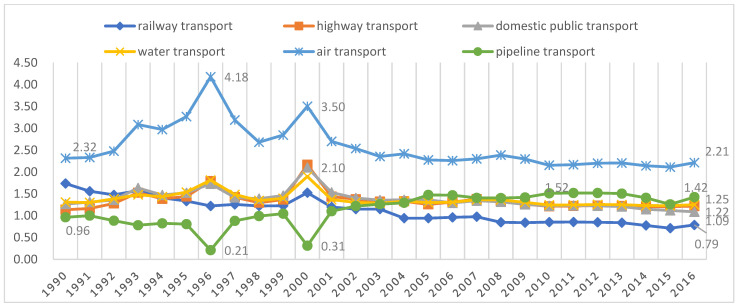
CO_2_ influence coefficient for the six transportation sectors over time.

The CO_2_ influence coefficient, i.e., the degree of the impact of each additional unit of final demand in each transport sector on CO_2_ emissions, is obtained with Equation (16). When the CO_2_ influence coefficient of a transport sector is greater than 1, it means that the transport sector’s impact on CO_2_ emissions exceeds the average of 123 sectors, and vice versa, it means that the transport sector’s impact on CO_2_ emissions is lower than the average of 123 sectors. Figure 1 reports the CO_2_ influence coefficient for each transport sector for the calendar years 1990–2016. Figure 1 shows that the CO_2_ influence coefficient of the pipeline transport sector was less than 1 for all years before 2001 except 1991 (influence coefficient of 1) and 1999 (influence coefficient of 1.04), while it was greater than 1 for each year from 2001 to 2016, reaching a maximum of 1.52 in 2011, i.e., from 2001 onward, the pipeline transport sector shifted from being a low-impact sector to a high-impact sector in terms of CO_2_ emissions. The influence coefficient for CO_2_ emissions from the air transport sector was greater than 1 for all 27 years and was as high as 4.18 in 1996. In addition, since China’s accession to the WTO, the CO_2_ influence coefficient of the railway sector has been declining from a maximum value of 1.53 in 2000 to 0.79 in 2016, dropping below 1 for the first time in 2004. In 2004, the railway transport sector shifted from being a high-impact sector in terms of CO_2_ emissions to being a low-impact sector. The remaining four transport sectors have had a consistently higher impact on CO_2_ emissions than the average across all sectors of the national economy over the 27-year period. However, after the U.S. subprime mortgage crisis, the CO_2_ influence coefficient for the highway, domestic public transport and water transport sectors slowly decreased to 1.22, 1.09 and 1.25, respectively, in 2016.

#### 3.1.2. Non-CO_2_ GHG Influence Coefficient

**Figure 2:**
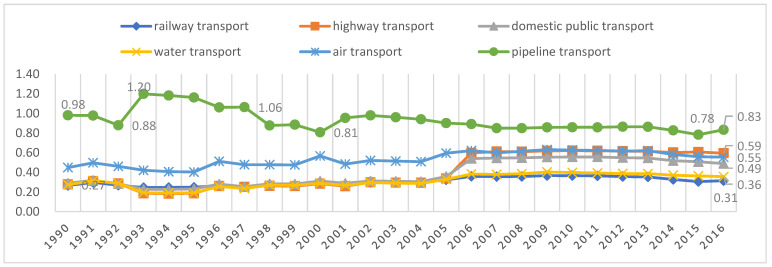
Non-CO_2_ GHG influence coefficient for the six transportation sectors over the years.

The non-CO_2_ GHG influence coefficient, i.e., the extent to which each one-unit increase in final demand in each transport sector affects non-CO_2_ GHG emissions, is obtained with Equation (16). Figure 2 reports the non-CO_2_ GHG emission impact coefficients for the six transportation sectors for the calendar years 1990–2016. Figure 2 shows that except for the pipeline transport sector, the influence coefficients of non-CO_2_ GHG emissions for the other five transport sectors are less than 1 in all years. The impact of the pipeline transport sector on non-CO_2_ GHG emissions was higher than the average across all sectors in society from 1993–1997 only, and during the other years, pipeline transport was also a low-impact sector in terms of non-CO_2_ GHG emissions. On the other hand, unlike the CO_2_ influence coefficient for each transport sector (except the pipeline transport sector), which declined slowly after the U.S. subprime mortgage crisis, the non-CO_2_ GHG influence coefficient for the six transport sectors tends to be constant after 2007 but to decline after 2013. The onset of the U.S. subprime mortgage crisis had no significant impact on the non-CO_2_ GHG emissions of the various transport sectors. The non-CO_2_ GHG influence coefficients for the six transport sectors in 2016 were 0.31, 0.59, 0.49, 0.36, 0.55, and 0.83.

### 3.2. GHG Emission Intensity Analysis

#### 3.2.1. CO_2_ Emission Intensity Analysis

**Figure 3:**
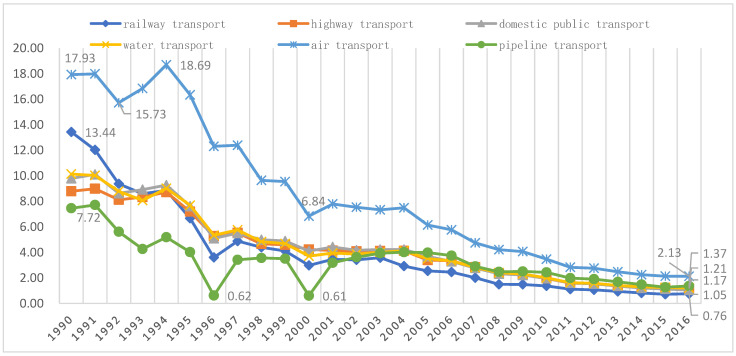
CO_2_ emission intensities of the six transportation sectors over time (kg/USD).

The complete CO_2_ emission coefficient, i.e., the total amount of CO_2_ that is emitted directly and indirectly for each additional unit of final demand in the transport sector, is obtained with Equation (9). That means the CO_2_ emission intensity obtained using the complete CO_2_ emission coefficient includes the entire process of the transportation sector from construction to operation. Figure 3 shows the CO_2_ emission intensities of the six transport sectors for all years between 1990 and 2016, while Table 2 shows the changes in the CO_2_ emission intensities of the six transport sectors over the four observation periods. Figure 3 shows that for the six transport sectors, the overall trend exhibits a significant decrease, except for a few years when their complete CO_2_ emission coefficients increase. Combined with the results in Table 2, it can be seen that the six transport sectors reduced their complete CO_2_ emission coefficients by 94.36%, 86.65%, 89.30%, 88.11%, 88.11%, and 81.62%, respectively, during these 27 years. Of the six sectors, the railway transport sector had the most significant reduction in CO_2_ emission intensity. The most significant reduction in CO_2_ emission intensity occurred in the railway transport sector from 1990–1994, with a reduction of 34.08%. The CO_2_ emission intensity in the air transport sector increased by 4.27% from 1990 to 1994 and peaked at 18.69 kg/USD in 1995. After China’s comprehensive market-oriented reform, its economy has grown rapidly, and the demand for transportation has continued to increase, but the CO_2_ emission intensity of the six transportation sectors has decreased significantly, with all of the transportation sectors excluding the pipeline transport sector maintaining a reduction of more than 50%. After China became a member of the WTO, the vigorous development of international trade further stimulated an increase in transportation demand, but the CO_2_ emission intensities of the six transportation sectors decreased further; except for the pipeline transport sector, the other five sectors exhibited decreases that exceeded 40%, with the decrease in the railway transport sector reaching as high as 56.49%. After the U.S. subprime mortgage crisis in 2008, China’s industrial structure was further optimized, and the per unit emissions of the six transportation sectors decreased by more than 45%. In 2016, the CO_2_ emission intensities of the six transportation sectors were 0.76, 1.17, 1.05, 1.21, 2.13, and 1.37 kg/USD.

#### 3.2.2. Non-CO_2_ GHG Emission Intensity Analysis

**Figure 4:**
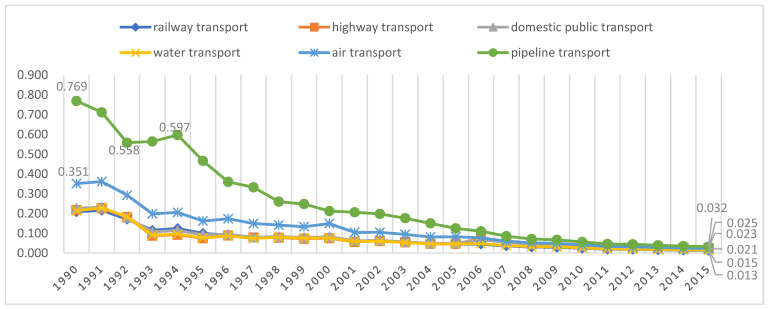
Non-CO2 GHG emission intensities of the six transportation sectors over time (kg/USD).

The complete non-CO_2_ GHG emission coefficient, i.e., the total amount of non-CO_2_ GHGs that are emitted directly or indirectly for each additional unit of final demand in the transport sector, is obtained with Equation (9). That means the non-CO_2_ emission intensity obtained using the complete non-CO_2_ emission coefficient includes the entire process of the transportation sector from construction to operation. Figure 4 shows the non-CO_2_ GHG emission intensities of the six transportation sectors for all years between 1990 and 2016, while Table 3 shows the changes in the non-CO_2_ GHG emission intensities of the six transportation sectors over the four observation periods. As shown in Table 3, the non-CO_2_ GHG emission intensities of the six transportation sectors decreased significantly by −94.44%, −89.92%, −92.05%, −93.87%, −94.20% and −96.01% from 1990 to 2016. The non-CO_2_ GHG emission intensity of the pipeline transport sector was much higher than those of the other transport sectors, and it has reduced emissions to a greater extent than the other transport sectors. The reduction in the non-CO_2_ GHG emission intensity of the pipeline transport sector exceeded 66% during both Observation Period 2 and Observation Period 3 and exceeded 50% during Observation Period 4. The non-CO_2_ GHG emission intensities for the six transportation sectors have been below 0.1 kg/USD since 1998. The non-CO_2_ GHG emission intensities for the six transportation sectors in 2016 were 0.013, 0.025, 0.021, 0.015, 0.023, and 0.032 kg/USD.

### 3.3. Impact of Transportation Restructuring on GHG Emission Intensity

#### 3.3.1. Impact of Transportation Restructuring on CO_2_ Emission Intensity

Table 4 demonstrates the extent to which transportation restructuring has affected the intensity of CO_2_ emissions in each sector. As Table 4 shows, although the CO_2_ emission intensity of each transport sector decreased significantly from 1990–2016, transportation restructuring both suppressed and increased the emission intensities during the different observation periods. The negative effect of transportation restructuring on CO_2_ emission intensity was significant in 1990–1994, as the CO_2_ emission intensities of the six transportation sectors decreased by 24.87%, 52.81%, 57.82%, 73.66%, 66.21%, and 50.98%, while transportation restructuring also reduced the CO_2_ emission intensities of the nontransportation sector by 5.79%.From 1994 to 2009, China’s transport restructuring increased the intensity of CO_2_ emissions from the six transportation sectors and the nontransportation sector. Among them, from 1994 to 2002, the CO_2_ emission intensity of water transportation and air transportation increased by 72.18% and 74.06% due to transportation restructuring; from 2002 to 2009, the transportation restructuring had a boosting effect of more than 20% on the CO_2_ emission intensity of both the six transportation sectors and the nontransportation sector. From 2009 to 2016, the emission intensities induced by China’s transportation restructuring is characterized by reductions in the CO_2_ emission intensities of the six transportation sectors of 12.71%, 13.01%, 13.76%, 15.23%, 18.10% and 17.95%, and the CO_2_ emissions intensity of the nontransportation sector also decreased by 16.76%.

#### 3.3.2. Impact of Transportation Restructuring on the Intensity of Non-CO_2_ GHG Emissions

Table 5 demonstrates the extent to which transportation restructuring affected the intensity of non-CO_2_ GHG emissions in each sector. Table 5 shows that the restructuring of the transportation industry increased the intensity of non-CO_2_ GHG emissions in each sector from 1994–2009 and decreased the intensity of non-CO_2_ GHG emissions in each sector from 1990–1994 and from 2009–2016, which are similar results to the ones in Table 4. However, from 1990 to 1994, the reduction in non-CO_2_ GHG emission intensity in the six transportation sectors due to transportation restructuring did not exceed 6.1%, while the reduction in non-CO_2_ GHG emission intensity in the nontransportation sector was only 0.08%. The increase in the emission intensity of each sector did not exceed 4% from 1994–2002, while the increase in emission intensity for each sector did not exceed 2% from 2002–2009, nor did the decrease in emission intensity for each transport sector exceed 1% from 2009–2016. 

## 4. Discussion

By calculating the CO_2_ and non-CO_2_ GHG influence coefficients for each transport sector, the impact of each transport sector on China’s GHG emissions can be quantitatively demonstrated to highlight the urgency of studying the impact of transportation restructuring on GHG emissions. 

Different from the traditional algorithm [45,46,47] for calculating GHG emission intensity, the complete GHG emission coefficient captures the GHG emissions directly and indirectly caused by each additional unit of final demand in each transport sector. Theoretically, the carbon emission of the transportation sector comes from both the construction stage and the operation stage. The GHG emission intensities obtained by calculating the complete GHG emission coefficient can more accurately show the per unit GHG emissions of each transport sector, including the construction stage and the operation stage.

The results in Table 2 and Table 3 suggest that the reductions in both the CO_2_ and non-CO_2_ GHG emission intensities in the railway transport sector from 1990–2016 were significant, reaching 94.36% and 94.44%, respectively. And Figure 1 and Figure 2 show that, since 2004, the railway transport sector has been a low-impact sector for both CO_2_ and non-CO_2_ GHG emissions, i.e., a low-impact sector for GHG emissions. China’s first high-speed railroad was officially opened to traffic in October 2003. Since then, the high-speed rail sector has developed rapidly. That illustrates the environmental effect of China’s vigorous development of its high-speed railway transportation system. This is consistent with Lane’s empirical findings [48].

Different from the conclusion of a linear relationship between transportation restructuring and GHG emission intensity obtained from the literature [16,17,18,19,20,21,22,23,24], this paper finds that the effect of transportation restructuring on GHG emission intensity is nonlinear, which is in line with Greening [28] and Wei [49]’s view. From 1990 to 1994, China was transitioning from a planned economy to a market economy, and both planned and market economies existed in the domestic market. Administrative documents had a greater influence on resource allocation. As a result, China’s transportation restructuring from 1990 to 1994 significantly reduced the intensity of CO_2_ emissions from the six transportation sectors and the nontransportation sector. However, after China’s comprehensive market economy reform, the demand for transportation increased considerably, and factors of production began to automatically flow to the low-cost and high-volume water and pipeline transport sectors, thus promoting the restructuring of the transportation industry, which in turn resulted in an increase in the CO_2_ emission intensities of the above two sectors by 72.18% and 74.06%, respectively, while the air transport sector also increased its CO_2_ emission intensity by 74.06%. The CO_2_ emission intensity increase in the nontransportation sector is caused by transportation restructuring at 14.18%. After China’s formal accession to the WTO, the rapid growth in international trade further boosted the demand for transport, and the spontaneous adjustment of the transportation structure at this stage occurred mainly to meet the increasing demand for transport without consideration of the impact of CO_2_ emissions on climate change. Therefore, from 2002 to 2009, the restructuring of the transportation industry increased the intensity of CO_2_ emissions in the six transportation sectors and the nontransportation sector, with the emission intensity of the air transport sector increasing by 35.35% and the emission intensities of the rest of the transport sectors increasing by more than 21%. 

As Fan and Lei [50] found, we find that China’s transportation structure was further optimized after the outbreak of the U.S. subprime mortgage crisis, and in line with the trend in the CO_2_ emission influence coefficient depicted in Figure 1, China’s transportation restructuring has again reduced CO_2_ emissions intensity in the six transportation sectors and the nontransportation sector. The post-2009 transportation restructuring significantly reduced the intensity of CO_2_ emissions in all sectors. There have been studies that found in both developing and developed countries, the health benefits of GHG emission reductions can offset most of the costs of abatement and even result in net benefits in some cases [51,52,53]. It is thus clear that China’s post-2009 transportation restructuring has provided a strong contribution to improving public health.

In addition, although the intensity of non-CO_2_ GHG emissions in each transport sector decreased significantly from 1990 to 2016, in none of the six transport sectors did the impact of transportation restructuring exceed 6.1%, and the impact on the intensity of non-CO_2_ GHG emissions in the nontransportation sectors did not exceed 1.3%. In summary, it is clear that the impact of transportation restructuring on the intensity of non-CO_2_ GHG emissions in all sectors was limited.

However, this study is not wholly beyond reproach, and indeed it has some weaknesses. Since the input-output tables provided in the EORA database are only updated to 2016, the impact of transportation restructuring on GHG emission intensity in recent years has not been studied in this paper. The EORA database provides data on the six non-CO_2_ GHG emissions specified in the Kyoto Protocol’s second commitment. Non-CO_2_ GHG emission data for each sector are difficult to obtain. To study the impact of transport restructuring on the CO_2_ and non-CO_2_ GHG emission intensity of each sector separately, this paper had to compromise on timeliness.

## 5. Conclusions and Implications

Reducing GHG emissions without compromising economic growth is a powerful step in the fight against climate change. Reducing GHG emissions in transportation is important for achieving deep decarbonization [11]. In this paper, the per unit CO_2_ and non-CO_2_ GHG emissions caused directly and indirectly by each transport sector are calculated separately by using a hybrid input-output model. Based on the dynamics of transportation restructuring in combination with the industrial evolution of China, the period 1990–2016 is divided into four observation periods. This paper uses noncompetitive input-output tables from 1990–2016 to study the impact of the restructuring of the Chinese transportation industry on the intensity of CO_2_ and non-CO_2_ GHG emissions in the six transportation sectors and the nontransportation sector. The main results are as follows. (1) The CO_2_ emission intensity and non-CO_2_ GHG emission intensity of all transportation sectors in China have been significantly reduced. (2) Although the intensity of GHG emissions has diminished across transport sectors, the highway, domestic public transportation, water and air transport sectors are still high-impact sectors for CO2 emissions. (3) The impact of transportation restructuring on the CO_2_ emission intensity of each transport sector and the nontransportation sector is different in different periods; specifically, transportation restructuring reduced the CO_2_ emission intensity of each transport sector and the nontransportation sector from 1990–1994 and 2009–2016 but increased the CO_2_ emission intensity of each transport sector and the nontransportation sector from 1994–2002 and 2002–2009. (4) The impact of transportation restructuring on the intensity of non-CO_2_ GHG emissions in all sectors was limited. (5) The railway transport sector transitioned from being a high-impact sector in terms of CO_2_ emissions to being a low-impact sector in 2004, and its emission intensity decreased to less than 1 kg/USD after 2012. The railway transport sector has become a low-impact sector in terms of GHG emissions. Interestingly, the pipeline transport sector, which used to be a high-impact sector for non-CO_2_ GHG emissions in 1993-1997, became a high-impact sector for CO_2_ emissions after 2001.

These findings have policy implications. First, to reduce GHG emissions, the relevant government departments should set stricter fuel economy standards [54] to promote the development of biofuel technology [55,56] and tax fuel to encourage technological innovation. As the transport sector with the greatest impact on CO_2_ emissions, air transport must continue to develop technologies such as electric aircraft to increase the electrification rate on the one hand and to vigorously develop hydrogen-driven and biofuel technologies on the other hand [36]. The water transport sector, which has the largest volume of freight, needs to continue to develop biofuel technology based on increased electrification rates [57]. Second, as seen from Table 4, the transportation restructuring that has taken place since 2009 has begun to effectively reduce the intensity of CO_2_ emissions in the six transportation sectors. The potential to reduce GHG emissions by optimizing the transportation structure is enormous. The railway transport sector has been a low-impact sector in terms of CO_2_ emissions since 2004, with a CO_2_ emissions intensity below 0.8 kg/USD and a non-CO_2_ GHG emissions intensity below 0.02 kg/USD in 2016, and is inherently characterized by high turnover and low costs. This gives the railway transport sector great potential for addressing GHG emission reductions [48]. All relevant government departments should earnestly implement The Outline of the National Comprehensive Three-dimensional Transportation Network Plan issued by the Chinese State Council to build a highly efficient, comprehensive, three-dimensional national transportation network to increase railway coverage and guide the continued shift of highway transport toward the railway transport sector. This will help China reduce GHG emissions and meet the goal of becoming carbon neutral by 2060.

## Figures and Tables

**Table 1:** The hybrid input-output structure.

	Input	Intermediate use				Final Use	Gross Output
Output		Sector 1	Sector 2	…	Sector n		
Intermediate Input	Sector 1	${\left(x_{\mathrm{ij}}\right)}_{nxn}$				y_i_	X_i_
	Sector 2						
	…						
	Sector n						
Value Added		v_j_					
Total Input		X_j_					
GHGs	CO_2_	g_1j_				$e_{1}$	$t_{1}$
	Non-CO_2_ GHG	g_2j_				$e_{2}$	$t_{2}$

**Table 2:** Changes in CO_2_ emission intensity in the transport sector, 1990–2016 (%).

	Railway	Highway	Domestic Public	Water	Air	Pipeline
1990–1994	−34.08	−0.78	−5.32	−11.02	4.27	−30.46
1994–2002	−61.41	−53.13	−54.99	−57.14	−59.69	−30.17
2002–2009	−56.49	−45.42	−46.65	−40.30	−46.08	−30.76
2009–2016	−49.04	−47.42	−52.92	−47.79	−47.53	−45.29
1990–2016	−94.36	−86.65	−89.30	−88.11	−88.11	−81.61

**Table 3:** Changes in non-CO_2_ GHG emission intensities in the transport sector, 1990–2016 (%).

	Railway	Highway	Domestic Public	Water	Air	Pipeline
1990–1994	−40.35	−57.48	−49.57	−55.45	−41.58	−22.36
1994–2002	−51.93	−34.85	−44.98	−37.63	−48.87	−66.90
2002–2009	−52.32	−20.13	−31.50	−47.37	−53.31	−66.29
2009–2016	−59.31	−54.45	−58.19	−58.12	−58.40	−53.94
1990–2016	−94.44	−89.92	−92.05	−93.87	−94.20	−96.01

**Table 4:** Impact of transportation restructuring on CO_2_ emission intensities (%).

	Railway	Highway	Domestic Public	Water	Air	Railway	Nontransportation
1990–1994	−24.87	−52.81	−57.82	−73.66	−66.21	−50.98	−5.79
1994–2002	23.03	46.48	33.94	72.18	74.06	45.40	14.18
2002–2009	22.91	28.08	21.04	28.96	35.35	29.30	26.22
2009–2016	−12.71	−13.01	−13.76	−15.23	−18.10	−17.95	−16.76

**Table 5:** Impact of transportation restructuring on non-CO_2_ GHG emission intensity (%).

	Railway	Highway	Domestic Public	Water	Air	Railway	Nontransportation
1990–1994	−2.46	−5.31	−6.05	−4.00	−4.77	−5.19	−0.08
1994–2002	1.68	2.97	2.67	3.44	3.73	3.70	0.66
2002–2009	1.11	1.14	1.00	1.11	1.38	1.45	1.29
2009–2016	−0.57	−0.56	−0.61	−0.63	−0.74	−0.81	−0.75

## Data Availability

Publicly available datasets were analyzed in this study. This data can be found here: https://www.worldmrio.com/ (accessed on 1 October 2022).
